# Escalation and De-Escalation of Antiplatelet Therapy after Acute Coronary Syndrome or PCI: Available Evidence and Implications for Practice

**DOI:** 10.3390/jcm11216246

**Published:** 2022-10-23

**Authors:** Felice Gragnano, Antonio Capolongo, Fabrizia Terracciano, Giuseppe Gargiulo, Vincenzo De Sio, Arturo Cesaro, Elisabetta Moscarella, Giuseppe Patti, Italo Porto, Giovanni Esposito, Dominick J. Angiolillo, Paolo Calabrò

**Affiliations:** 1 Department of Translational Medical Sciences, University of Campania “Luigi Vanvitelli”, 80131 Naples, Italy; 2 Division of Cardiology, Azienda Ospedaliera Sant’Anna e San Sebastiano, 81100 Caserta, Italy; 3 Department of Advanced Biomedical Sciences, Federico II University of Naples, 80131 Naples, Italy; 4 Department of Translational Medicine, University of Eastern Piedmont, 28100 Novara, Italy; 5 Division of Cardiovascular Medicine, Policlinico San Martino, University of Genova, 16132 Genova, Italy; 6 Division of Cardiology, University of Florida College of Medicine, Jacksonville, FL 32209, USA

**Keywords:** de-escalation, escalation, DAPT, guided-therapy, aspirin, P2Y12 inhibitors

## Abstract

Dual antiplatelet therapy (DAPT) is the gold standard for the antithrombotic management of patients with an acute coronary syndrome (ACS) or undergoing percutaneous coronary intervention (PCI). Implementation of intensified or prolonged DAPT regimens has proven to lower the risk of ischemic events but at the expense of increased bleeding. Importantly, bleeding is a predictor of poor prognosis. Risk stratification and selection of tailored antiplatelet strategies to maximize the net clinical benefit in individual patients with ACS or undergoing PCI is therefore potentially beneficial. Recently, novel approaches including DAPT de-escalation or escalation have been proposed as possible alternatives to standard DAPT. These strategies, which are generally based on patient’s risk profile, genetics, and/or platelet function have been proposed to offer more tailored treatments in patients with ACS or PCI, with the ultimate goal of providing adequate ischemic protection while mitigating the risk of bleeding. This review summarizes the available evidence on DAPT de-escalation or escalation (both guided and unguided) and discusses the practical implications of these strategies in the contemporary management of patients with ACS and/or undergoing PCI.

## 1. Introduction

Dual antiplatelet therapy (DAPT) remains a cornerstone in the management of patients with acute coronary syndrome (ACS) or undergoing percutaneous coronary intervention (PCI) [1,2,3,4,5,6,7,8,9]. Substantial evidence supports the use of DAPT in reducing the risk of thrombotic events by acting on two key pathways of platelet activation and aggregation (i.e., the inhibition of cyclooxygenase-1 and platelet P2Y12 receptors). In patients with ACS, current guidelines recommend 12 months of DAPT with aspirin and an oral P2Y12 receptor inhibitor (preferably ticagrelor or prasugrel over clopidogrel) [3]. After elective PCI, DAPT with aspirin and clopidogrel is recommended for 6 months [2]. DAPT duration is usually tailored to the individual risk: if patients are deemed at high bleeding risk, then DAPT duration can be shortened by up to 1 month in the case of both ACS or elective PCI [1,10,11]; conversely, in case of low bleeding risk and high ischemic risk, DAPT can be prolonged beyond 12 months [1,12,13]. Recently, alternative approaches have been proposed, including strategies for DAPT escalation and de-escalation [14]. These strategies, which are generally tailored according to the patient’s risk profile, genetics, or platelet function have been proposed to individualize treatment and mitigate both ischemic and bleeding risks in patients with ACS and/or undergoing PCI [15,16,17,18]. 

## 2. Oral P2Y12 Receptor Inhibitors: Pharmacokinetics and Pharmacodynamics

**Figure 1:**
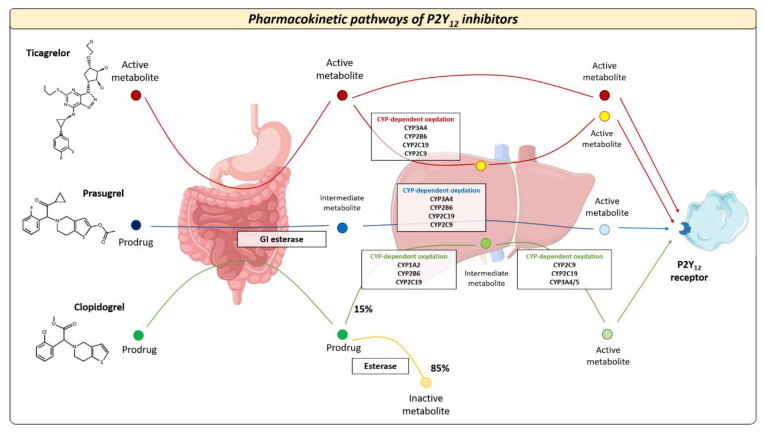
Pharmacokinetic pathways of oral P2Y12 inhibitors. After intestinal absorption, oral P2Y12 inhibitors exhibit different metabolic pathways that affect their antiplatelet effects. CYP = cytochrome P450.

**Figure 2:**
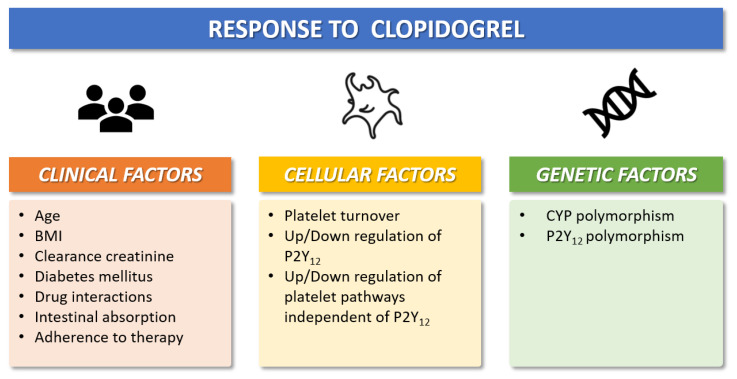
Clinical, cellular, and genetic factors that modulate individual antiplatelet response to clopidogrel. BMI = Body-mass-index; CYP = cytochrome P450.

The pharmacokinetic and pharmacodynamic features of available oral P2Y12 inhibitors vary widely across different agents. Oral P2Y12 receptor inhibitors can be classified as direct inhibitors (i.e., ticagrelor) or pro-drugs (i.e., clopidogrel and prasugrel) [15,16,17,18]. Clopidogrel is the most commonly used P2Y12 inhibitor 2. Following oral ingestion of clopidogrel, approximately 85% of gastrointestinal absorbed pro-drug is inactivated by plasma esterases, whereas the remaining 15% undergoes two sequential oxidations before its transformation into the active metabolite [15,16] (Figure 1). These processes are mediated by the cytochrome CYP2C19, which has different allelic variants that identify five clinical phenotypes: poor metabolizer (PM), intermediate metabolizer (IM), normal metabolizer (NM), rapid metabolizer (RM), and ultrarapid metabolizer (UM) [17]. Prasugrel is also a pro-drug but requires only one-step oxidation to form its active metabolite. Ticagrelor is a direct P2Y12 inhibitor and does not require hepatic biotransformation, although 30% of the effects of the drug are attributed to a CYP3A4-derived metabolite [17]. The antiplatelet effects of prasugrel and ticagrelor are not related to CYP2C19 polymorphisms and randomized trials showed the superiority of both drugs versus clopidogrel after ACS at the expense of a higher risk of bleeding [19]. The superior efficacy of prasugrel and ticagrelor is determined by higher intrinsic antithrombotic effect, wide interindividual variability in CYP gene expression, and clinical determinants that can influence the response to clopidogrel in patients with ACS (Figure 2): about 10–40% of the general population (with proportions that vary across ethnicities) are known to be carriers of Loss-of-Function (LoF) alleles of CYP2C19, which results in a loss of enzymatic function and, therefore, in reduced activation of the pro-drug [15,16,17,18]. The presence of allelic variants causes loss of cytochrome function and decreased production of the active clopidogrel metabolite (i.e., CYP2C19 * 2 and * 3), which in turn results in a high platelet reactivity (HPR) status during clopidogrel treatment; this condition is associated with a greater risk of ischemic events [14]. Conversely, prasugrel and ticagrelor have a more predictable antiplatelet effect, with an incidence of on-treatment HPR of less than 5% [15,16,17,18,20].

## 3. Platelet Function and Genetic Tests to Guide Antiplatelet Therapy

Platelet function tests allow for the assessment of platelet reactivity and identification of HPR status in patients with ACS or PCI [15,16,17,18]. Available tests are based on various methods, including (i) assessment of platelet aggregation, (ii) evaluation of platelets under specific stress conditions, (iii) platelet plug analysis, and (iv) measurements of factors released from the activated platelets (Table 1) [15,16,17,21,22,23]. Some tests can be performed at the bedside (i.e., point-of-care assays), therefore making them easy to perform and time-saving. A major drawback of platelet function tests is the need to perform the test while the patient is on treatment with an antiplatelet agent, ideally during the steady state phase once full effects have been achieved, for which the therapeutic response is tested [15]. Among these tests, light transmission aggregometry (LTA) is considered the gold standard for platelet function evaluation and allows the estimation of residual platelet reactivity (an index that can be used to predict the risk of major cardiovascular events in patients with coronary atherosclerosis) [15,16,17]. The Multiplate^®^ platelet function analyzer provides an easy and accurate evaluation of platelet function in response to specific agonists (i.e., adenosine diphosphate [ADP], arachidonic acid, thrombin receptor activator peptide [TRAP]-6) [15,23]. The VerifyNow can measure platelet aggregation induced by an exogenous agonist (i.e., ADP, arachidonic acid) and has different laboratory assays to evaluate the effect of glycoprotein IIb/IIIa antagonists, aspirin (expressed in aspirin reaction units [ARUs]), and thienopyridines (expressed in P2Y12 reaction units [PRUs]) [22]. The platelet function analyzer (PFA)-100 evaluates platelet adhesion under specific shear stress conditions, and it is performed using specific types of cartridges: the CADP (with collagen and ADP), CEPI (with collagen and epinephrine), and INNOVANCE P2Y (with ADP and prostaglandin E1) [15,16,17,18]. 

Genetic tests have several potential advantages compared with platelet function tests, including the possibility to be performed at any time and irrespective of the use of antiplatelet therapy, providing information that does not change with time (Table 2) [15,16,17,18]. The Spartan RX (Spartan Bioscience Inc., Ottawa, Canada) is a rapid and accurate test that is able to define the CYP2C19 allele status (*1, *2, *3, *17) within 1 h [17,24,25]. The Verigene (Nanosphere Inc., Northbrook, Illinois, US) uses a fully automated microarray that rapidly identifies twelve allelic variants of CYP2C19 (*1–*10, *13, *17) [26] showing a concordance of nearly 100% to DNA sequencing methods [17,25]. The ST Q3 (STMicroelectronics, Geneva, Switzerland) system uses a real-time polymerase chain reaction to define the genotype [27,28]. This is a point-of-care and low-cost system that requires about 70 min for data processing, but it requires further validation studies for clinical use [27,28]. Although genetic testing can help predict the response to antiplatelet drugs, these tests provide only part of the information, as other determinants, such as clinical factors, also contribute to the antiplatelet drug response [29].

Both platelet function and genetic tests can be used to guide antiplatelet therapy. Yet, genetic tests appear more suitable for routine application in clinical practice as they can be performed in the peri-procedural setting (i.e., during the hospitalization for ACS or PCI) [15,16,17,18]. At variance, platelet function tests may require additional post-discharge hospital visits to test therapeutic response after 1–2 weeks of treatment, which may complicate their routine implementation. Of note, utilization of both techniques increases the time and costs for the management of PCI patients and requires adequate expertise for interpretation of results [15,16,17,18]. These aspects represent potential barriers for their routine use in unselected patients with ACS or PCI in daily practice.

### 3.1. Escalation and De-Escalation of DAPT after ACS or PCI

**Figure 3:**
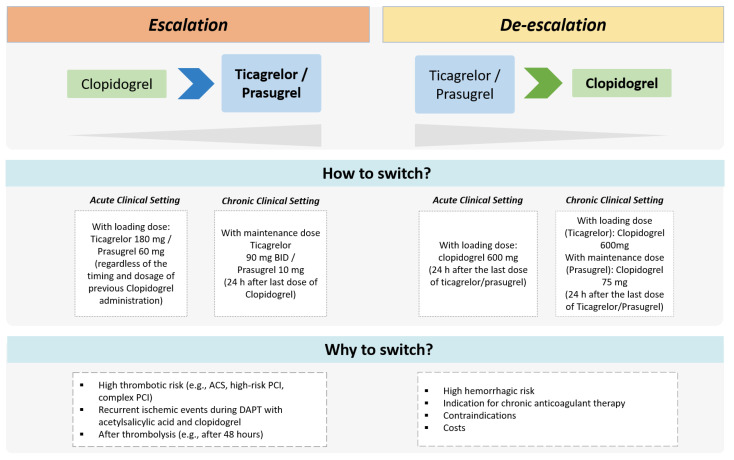
Therapeutic approaches of escalated and de-escalated DAPT. ACS = acute coronary syndrome; BID = bis-in-die; PCI = percutaneous coronary intervention.

Novel treatment strategies of DAPT escalation and de-escalation have been evaluated recently in randomized trials showing potential advantages compared with standard DAPT regimens (Figure 3) [15,16,17,18]. The escalation consists of switching from a less potent (i.e., clopidogrel) to a more potent oral P2Y12 inhibitor (i.e., ticagrelor or prasugrel) with the goal of reducing thrombotic risk after ACS and/or PCI. At variance, the de-escalation consists of switching from ticagrelor or prasugrel to a reduced dosing regimen or switching to clopidogrel in order to decrease bleeding risk [15,30]. Both escalation and de-escalation approaches can be guided or unguided (Table 3). The guided approach involves the use of genetic or platelet function tests to identify carriers of LoF alleles of CYP2C19 or assess the HPR status, respectively. The unguided approach involves escalating or de-escalating DAPT based on clinical considerations only (i.e., not informed by genetic or platelet function tests).

### 3.2. Available Evidence for a Guided Approach Using Platelet Function Tests

The ADAPT-DES trial [31] was a prospective, multicenter registry that included patients undergoing PCI with drug-eluting stents treated with aspirin plus clopidogrel. The study examined the relation between on-treatment platelet reactivity (measured with VerifyNow point-of-care assays) and one-year clinical outcomes. Among 8583 patients who were eligible for analysis, HPR on clopidogrel was associated with higher risks of stent thrombosis (adjusted hazard ratio [HR]: 2.49, 95% CI 1.43–4.31; *p* = 0.001) and myocardial infarction (adjusted HR: 1.42, 95% CI: 1.09–1.86, *p* = 0.01), and lower risk of bleeding (adjusted HR: 0.73, 95% CI: 0.61–0.89; *p* = 0.002) but was not related to death. HPR on aspirin was not significantly associated with ischemic or fatal events, but showed significant inverse association with bleeding (adjusted HR: 0.65, 95% CI: 0.43–0.99; *p* = 0.04).

The GRAVITAS trial [32] evaluated the effects of a high dose versus a standard dose of clopidogrel among patients with high on-treatment platelet reactivity after PCI. Overall, 2214 patients with high on-treatment reactivity 12–24 h after PCI were randomized to receive high-dose clopidogrel (600 mg loading dose, 150 mg/day thereafter) or standard-dose clopidogrel (no additional loading dose, 75 mg/day). After 6 months, the primary endpoint of cardiovascular death, myocardial infarction (MI), or stent thrombosis occurred in 2.3% of patients on high-dose clopidogrel and 2.3% of patients on standard-dose clopidogrel (HR: 1.01, 95% CI: 0.58–1.76; *p* = 0.97). Severe or moderate bleeding was not increased with the high-dose regimen (1.4% vs. 2.3%; HR: 0.59, 95% CI: 0.31–1.11; *p* = 0.10). Compared with standard-dose clopidogrel, high-dose clopidogrel provided a 22% absolute reduction in the rate of high on-treatment reactivity at 30 days (62% vs. 40%, 95% CI: 37–43%; *p* < 0.001).

In the randomized TRIGGER-PCI [33] trial, the investigators evaluated the possible benefits of DAPT escalation from clopidogrel to prasugrel after elective PCI in selected patients with HPR (i.e., PRU > 208). Among 212 participants receiving prasugrel, PRU decreased from a median of 245 (225–273) at baseline to 80 (42–124) after 3 months; in 211 patients who were assigned to clopidogrel, PRU decreased from 249 (225–277) to 241 (194–275). These results provided evidence of a significant difference in PRU reduction, favoring prasugrel over clopidogrel (*p* < 0.001). However, the study was prematurely stopped because of the low event rates, remaining inconclusive for clinical outcomes [31].

In the ANTARCTIC trial [34], 877 patients with ≥75 years undergoing coronary stenting for ACS were randomized to receive prasugrel 5 mg/day with dose or drug adjustment in case of inadequate response (monitoring group) or prasugrel 5 mg/day with no monitoring or treatment adjustment (conventional group). Platelet function was assessed 14 days after randomization and then repeated 14 days after switching the dose of prasugrel in the experimental group. The primary endpoint of cardiovascular death, MI, stroke, stent thrombosis, urgent revascularization, and Bleeding Academic Research Consortium (BARC) 2, 3, or 5 bleeding at 1 year occurred in 28% of cases in both groups (HR: 1.00, 95% CI: 0.78–1.29; *p* = 0.98), with no differences in terms of bleeding events [34].

In the TROPICAL-ACS trial [35], an early guided de-escalation (from prasugrel to clopidogrel) was compared with a standard DAPT in 2610 patients with ACS receiving PCI. In the experimental group, 1304 patients were treated with DAPT including prasugrel for the first 7 days after PCI and clopidogrel from day 7 to day 14. On day 14, platelet function tests were performed and patients were switched back to prasugrel if HPR or continued with clopidogrel if not HPR. In the control group, 1306 patients were treated with a standard DAPT of aspirin and prasugrel for 12 months. The HPR status was documented in 511 patients (39%) in the experimental group, of whom 506 were switched to prasugrel. At 1 year, the primary endpoint of net clinical benefit (a composite of cardiovascular death, MI, stroke, and BARC 2, 3, or 5 bleeding) occurred in 7% of cases in the experimental group and 9% in the control group, showing the non-inferiority of de-escalation versus standard DAPT (P-non-inferiority = 0.0004; HR: 0.81, 95% CI: 0.62–1.06). Of note, the risk of secondary ischemic endpoints was not different between the experimental and control groups [35].

In the PATH-PCI trial, an experimental strategy of guided escalation was compared with a reference strategy of standard DAPT among 2237 patients receiving elective PCI [36]. In the experimental group, the choice of the P2Y12 inhibitor was based on the maximum aggregation rate (MAR) index determined via the PL-12 testing: in the case of MAR above 55%, ticagrelor was prescribed; conversely, DAPT with clopidogrel was chosen. At 180 days of follow-up, the primary endpoint of cardiac death, MI, stroke, stent thrombosis, urgent revascularization, and BARC 2, 3, or 5 bleeding was reduced with the experimental strategy compared with the reference strategy (5.1% vs. 7.5%; HR: 0.67, 95% CI: 0.48–0.94, *p* = 0.023) suggesting that an individualized approach based on the MAR index can improve the net clinical benefit after PCI [36].

### 3.3. Available Evidence for a Guided Approach Using Genetic Tests

In the POPular Genetics trial [37], a genetic-guided antiplatelet therapy was compared with standard DAPT in 2488 patients undergoing primary PCI for ST-segment elevation myocardial infarction. In the experimental group, after genetic assessment, carriers of CYP2C19 LoF alleles received DAPT with ticagrelor or prasugrel, whereas those without LoF alleles were treated with clopidogrel. In the control group, patients received standard DAPT, with ticagrelor or prasugrel given in 93% of cases. The primary endpoint of net clinical benefit at 1 year (a composite of death, MI, stroke, and major bleeding according to Platelet Inhibition and Patient Outcomes [PLATO] criteria) occurred in 5.1% and 5.9% of cases in the experimental and control groups, respectively (absolute difference: −0.7%; 95% CI: −2.0–0.7; P-non-inferiority < 0.0001). The incidence of major or minor bleeding was lower in the experimental arm than in the control arm (9.8% vs. 12.5%; HR: 0.78; 95% CI: 0.61–0.98; *p* = 0.04).

The PHARMCLO trial28, which was prematurely stopped after the inclusion of 888 patients, was designed to compare genetic-guided versus standard antiplatelet therapy after ACS. In the experimental group, the selection of the P2Y12 inhibitor was based on both clinical characteristics plus the results of genetic tests, which included the genotyping of ABCB1, CYP2C19 * 2, and CYP2C19 * 17 using ST Q3 system. In the control group, the selection of the P2Y12 inhibitor was based on clinical characteristics alone. Clopidogrel was used more often in the control group, ticagrelor in the experimental group, and prasugrel similarly in both groups. The rate of the primary endpoint at 1 year (a composite of cardiovascular death, MI, stroke, and BARC 3 or 5 bleeding) was significantly lower in the genetic-guided group than in the control group (15.9% vs. 25.9%; HR: 0.58; 95% CI: 0.43–0.78; *p* < 0.001) [28].

In the TAILOR-PCI trial [38], 5302 patients undergoing either elective or urgent PCI were included (82% with ACS). Patients in the experimental group (N = 2652) received the “point-of-care” Spartan Rx test to inform the antiplatelet therapy: CYP2C19 LoF carriers received ticagrelor, whereas non-carriers received clopidogrel. Patients randomized in the control group (N = 2650) received clopidogrel and underwent genotyping only after 12 months. Of 1849 patients with CYP2C19 LoF variants, 764/903 patients (85%) assigned to genotype-guided therapy received ticagrelor, and 932/946 patients (99%) assigned to standard DAPT received clopidogrel. At 1 year of follow-up, the primary endpoint of cardiovascular death, MI, stroke, stent thrombosis, and severe recurrent ischemia occurred in 4% of CYP2C19 LOF carriers in the experimental group and 5.9% in the control group, which however did not reach statistically significance (HR: 0.66; 95% CI: 0.43–1.02; *p* = 0.06). 

The ADAPT-PCI trial [39] was a randomized controlled trial investigating the clinical implications of CYP2C19 genotyping for antiplatelet therapy selection after stent implantation. The primary endpoint was the proportion of patients receiving prasugrel or ticagrelor in each arm. A total of 504 participants were randomized to point-of-care CYP2C19 genotyping (N = 249) or usual care (N = 255). In the genotyped group, 28% of patients carried CYP2C19 LoF alleles. Ticagrelor or prasugrel was prescribed significantly more often in the genotyped group than in the usual care group (30% vs. 21%; OR: 1.60; 95% CI: 1.07–2.42; *p* = 0.03). Significantly more CYP2C19 LoF carriers versus non-LoF carriers (53% vs. 22%) were prescribed prasugrel or ticagrelor. The choice of the P2Y12 inhibitor was guided by genetic tests but also by clinical considerations, including clinical presentation [39]. There were no differences between the genotyped and usual care groups for major adverse cardiovascular events (13.7% vs. 10.2%) and major bleeding (2.4% vs. 3.1%).

### 3.4. Available Evidence for the Use of Unguided Approaches

The TOPIC [40] trial enrolled 645 patients with ACS who received DAPT with ticagrelor or prasugrel and were free from events 1 month after PCI; they were randomized to an unguided de-escalation with aspirin plus clopidogrel (switched DAPT group) or a standard therapy of aspirin plus ticagrelor (unchanged DAPT). At 1 year, the primary composite endpoint of ischemic and bleeding events (including cardiovascular death, urgent revascularization, stroke, and BARC ≥ 2 bleeding) was significantly lower in the switched DAPT group than in the unchanged DAPT group (13.4% vs. 26.3%; HR: 0.48; 95% CI: 0.34–0.68; *p* < 0.01). Unguided de-escalation was also associated with a reduction in major or clinically relevant bleeding (HR: 0.30; 95% CI: 0.18–0.50; *p* < 0.01) [40]. 

The HOST-REDUCE POLYTHEC-ACS study randomized 2338 patients with ACS who underwent PCI and received DAPT with prasugrel 10 mg/day for the first month, to a de-escalation group (N = 1170) in which prasugrel was reduced to 5 mg/day, and a control group (N = 1168) in which prasugrel was continued at 10 mg/day 41. At 1-year follow-up, the primary endpoint of net clinical benefit (a composite of death, MI, stent thrombosis, multiple revascularizations, stroke, and major/clinically relevant bleeding) was significantly reduced in the de-escalated group compared to the control group (7.2% vs. 10.1%; HR: 0.70, 95% CI: 0.52–0.92; *p* = 0.012). Moreover, in the de-escalated group, there was no evidence of an increased risk of ischemic events (HR: 0.40, 95% CI: 0.40–1.45; *p* = 0.40) and bleeding events were significantly reduced (HR: 0.48, 0.32–0.73; *p* = 0.0007) [41].

The TALOS-MI trial [42] included 2697 East Asian patients with MI receiving DAPT with ticagrelor and free from ischemic and bleeding events at 1 month, in order to compare a standard DAPT with ticagrelor (N = 1348) versus a de-escalation strategy with clopidogrel (N = 1349) after the first month. At 1 year, the primary endpoint of cardiovascular death, MI, stroke, and BARC bleeding ≥ 2 was significantly reduced in the de-escalation group compared to the standard DAPT group (HR: 0.55, 95% CI: 0.40–0.76; *p* < 0.001). Furthermore, the composite safety endpoint, which included BARC bleeding ≥ 2, was significantly reduced in the de-escalated group compared with controls (HR: 0.52, 95% CI: 0.35–0.77; *p* = 0.0012) [42].

### 3.5. Pooled Evidence for the Use of Guided or Unguided Escalation/De-Escalation Strategies 

Several meta-analyses have been conducted to further assess the effects of escalating or de-escalating DAPT after ACS or PCI. In 2021, a meta-analysis of eleven randomized trials and three observational studies (N = 20,743) evaluated the effect of guided versus standard antiplatelet therapy after PCI [43]. Guided antiplatelet therapy was associated with a significant reduction in major cardiovascular events (risk ratio [RR]: 0.78, 95% CI: 0.63–0.95; *p* = 0.015) and a numerical reduction in bleeding (RR: 0.88, 0.77–1.01; *p* = 0.069). Secondary endpoints including cardiovascular death, MI, stroke, stent thrombosis, and minor bleeding were also significantly reduced with the guided strategy. Treatment effects were modulated by the type of approach, showing significant reductions in ischemic events with a guided escalation and reductions in bleeding events with guided de-escalation [43]. In 2022, a subsequent network meta-analysis of fifteen randomized trials of patients with ACS (N = 61,898) evaluated the efficacy and safety of a guided approach versus standard DAPT44. This study showed that a guided approach was superior in the prevention of major cardiovascular events (IRR [incidence rate ratios]: 0.80, 95% CI: 0.65–0.98) without a trade-off in terms of bleeding (IRR: 1.22, 0.96–1.55) [44]. Another meta-analysis of nineteen randomized trials (N = 69,746) compared guided versus unguided de-escalation after ACS45. In this analysis, an unguided de-escalation was associated with a lower risk of the primary outcome of major or minor bleeding compared with a guided strategy (HR: 0.48, 95% CI: 0.33–0.72), with no evidence of an increased risk of major cardiovascular events (HR: 0.82, 95% CI: 0.53–1.28). These results were consistent when the guided strategy was stratified according to the type of test (i.e., platelet function tests or genetic assessment) [45]. 

In general, the above-mentioned meta-analyses pooled data from contemporary and non-contemporary trials, in some cases with a low prevalence of drug-eluting stents, and trials including patients with different risk profiles [43,44,45]. A more recent meta-analysis of randomized trials including exclusively ACS patients undergoing implantation of current-generation drug-eluting stents, compared to the effect of DAPT de-escalation (both guided and unguided) with standard DAPT with ticagrelor or prasugrel [46]. This study showed that de-escalating DAPT can reduce bleeding by 43% (odds ratio [OR]: 0.57, 95% CI: 0.40–0.80, *p* = 0.001) and can improve the net clinical benefit endpoint by 41% (OR: 0.59, 95% CI: 0.41–0.85, *p* = 0.004) without increasing ischemic risk [46].

## 4. Conclusions

Available evidence indicates that the use of escalation or de-escalation strategies, both guided and unguided, improves the prognosis of patients with ACS and/or undergoing PCI. Current guidelines recommend the use of genetic or platelet function tests only in selected cases, reserving the choice of the type of test to physicians (Table 4) [3,8]. In keeping with these recommendations, guided antiplatelet strategies might be particularly useful in specific patients who are expected to benefit more from a tailored treatment (i.e., those with concomitantly high risks of ischemic and bleeding complications, or with recurrent events during DAPT). Notably, recent evidence also indicates the safety and efficacy of unguided approaches, which may be intuitively easier to apply in clinical practice and cost-effective compared with guided approaches, as they do not require any additional visits or tests. More randomized trial data are needed to more accurately establish patient cohorts that benefit most from guided versus unguided antiplatelet strategies in terms of ischemic protection and bleeding prevention. The use of tailored antiplatelet strategies represents a step towards a precision medicine approach in the antithrombotic management of patients with coronary artery disease with the potential to be implemented routinely in clinical practice [47].

## Figures and Tables

**Table 1:** Platelet function tests to guide antiplatelet therapy.

	Parameters Assessed	Methods	Advantages	Disadvantages
**Tests based on platelet aggregation**	**Light transmission aggregometry (LTA)**			
	Phases of platelet activation: shape modification, aggregation, and degranulation.	It measures the increase in light transmission through a plasma-rich suspension (platelet rich plasma [PRP]) after the addition of exogenous agonist factors (ADP, collagen, arachidonic acid and epinephrine).	Gold standard: flexibility, independence with respect to agonist concentration, ability to study different platelet pathways.	Time-consuming, it requires high expertise. It is also affected by variability in PRP preparation; type of agonists used; haemolysis; low platelet levels
	**Impedance whole blood aggregometry (WBA); Multiplate**			
	Platelet aggregation and monitoring the efficacy of antiplatelet therapy.	It measures the impedance change resulting from platelet aggregation in response to various exogenous agonist factors (ADP, arachidonic acid, TRAP6).	Whole blood test; easy availability of test.	It is influenced by hematocrit values and platelet count, concomitant use of anticoagulants, and time from sample collection to platelet test.
	**VerifyNow**			
	Platelet aggregation in responding to antiplatelet drugs: GpIIb/IIIa antagonists; ASA; thienopyridines.	Turbidimetric optical method that evaluates the increase in light transmission through the blood sample for the platelet aggregation induced by fibrinogen and platelet agonists (ADP, arachidonic acid).	Highly standardized, requiring no blood manipulation.	It is influenced by fibrinogen levels, hematocrit values, platelet counts, triglyceride levels, and the time between sample collection and test performance.
**Tests evaluating platelet adhesion under shear stress conditions**	**Platelet function analyzer (PFA-100)**			
	Adhesion and aggregation during platelet plug formation.	A blood sample is drawn through a system consisting of a membrane coated with platelet agonists until the gap in the membrane is occluded by the platelet plug.	Whole blood test; simple; rapid; small blood volumes needed as sample.	It is influenced by hematocrit values, platelet count, dependent on VWF levels.
**Tests based on platelet function in combination with viscoelastic tests**	**Thromboelastography (TEG o ROTEM)**			
	Haemostatic function, from thrombus formation to lysis.	A blood sample is put into a cylindrical cup and swung until a clot is formed.	Whole blood testing; performing “point-of-care” to monitor antiplatelet therapy.	It exclusively measures clot properties.
**Tests based on** **thromboxane metabolites**	**Radium/enzyme-related immunoassay**			
	Platelet activation status.	Thromboxane metabolism assay on serum or urine.	Dependent on platelet COX-1 enzyme.	Numerous artefacts.

**Table 2:** Genetic testing to guide antiplatelet therapy.

SPARTAN RX (Spartan Bioscience Inc., Ottawa, Canada)		ADVANTAGES	DISADVANTAGES
METHOD	TIME, min		
Four steps (performed in less than 8 min): - acquisition of a buccal swab; - insertion of the swab into the cartridge; - insertion of the reaction solution into the device; - analysis of CYP2C19 genotype by the device.	60	1. Absence of assay-dependent variability. 2. Independent of current therapy. 3. Time-invariant response. 4. Independent of results from non-patient-related factors.	1. Do not recognize all CYP2C19 enzyme polymorphisms, although rare. 2. Lack of availability in many centers.
**VERIGENE (Nanosphere Inc., Northbrook, Illinois, US)**			
**METHOD**	**TIME, min**		
It makes use of a microarray that identifies 12 allelic variants of CYP2C19 (*1–*10, *13 and *17) through a few steps: - venous sampling; - use of a disposable cartridge containing the array slide; - use of the hybridization reagents; - insertion into the Verigene Processor SP (Nanosphere).	180		
**ST Q3 SYSTEM** **(STMicroelectronics, Geneva, Switzerland)**			
**METHOD**	**TIME, min**		
It makes use of a “lab-on-chip” that provides genotyping by RT-PCR: - peripheral blood sampling; - DNA isolation through the Maxwell16TM platform; - loading of purified DNA into the ST lab-on-chip; - insertion of the chip into the ST Q3 instrument; - sample analysis and visualization of results.	70		

**Table 3:** Randomized clinical trials that evaluated DAPT escalation or de-escalation strategies (guided or unguided).

Test	Strategy	Study	Year	Patients Enrolled	Clinical Presentation	Follow-Up	Experimental Arm vs. Control	Primary Endpoint	Primary Endpoint Reached?
**PLATELET** **FUNCTION** **TEST**	**ESCALATION**	**PATH-PCI**	2019	2285	ACS: 0% CCS: 100%	6 mo.	Ticagrelor in patients nonresponders to clopidogrel vs. standard therapy.	Cardiovascular death, MI, stent thrombosis, urgent revascularization, bleeding BARC 3–5	Yes
		**TRIGGER-PCI**	2012	423	ACS: 0% CCS: 100%	6 mo.	Prasugrel vs. clopidogrel in clopidogrel nonresponders.	Cardiovascular death or MI	No
	**DE-** **ESCALATION**	**ANTARCTIC**	2016	877	ACS: 100% CCS: 0%	12 mo.	Therapy guided by platelet function testing vs. standard DAPT.	Cardiovascular death, MI, stent thrombosis, urgent revascularization stroke, bleeding BARC 2–5	No
		**TROPICAL-ACS**	2017	2610	ACS: 100% CCS: 0%	12 mo.	Therapy guided by platelet function testing vs. standard DAPT.	Cardiovascular death, MI, stroke, bleeding BARC 2–5	Yes
**GENETIC** **TESTING**	**ESCALATION**	**ADAPT**	2020	504	ACS: 50% CCS: 50%	16 mo.	Prasugrel or ticagrelor in patients nonresponders to clopidogrel vs. standard therapy.	Cardiovascular death, MI, urgent revascularization, stent thrombosis	No
		**PHARMCLO**	2018	888	ACS: 97% CCS: 3%	12 mo.	Prasugrel or ticagrelor in patients nonresponders to clopidogrel vs. standard therapy.	Cardiovascular death, MI, stroke, bleeding BARC 3–5	Yes
		**TAILOR-PCI**	2020	5302	ACS: 69% CCS: 31%	12 mo.	Prasugrel or ticagrelor in patients nonresponders to clopidogrel vs. standard therapy.	Cardiovascular death, MI, stroke, stent thrombosis, recurrent severe ischemia	No
	**DE-** **ESCALATION**	**POPular Genetics**	2019	2488	ACS: 100% CCS: 0%	12 mo.	Genotype-driven de-escalation vs. standard DAPT.	Death from all causes, MI, stent thrombosis, stroke, major bleeding according to PLATO criteria	Yes
**UNGUIDED** **APPROACH**	**DE-** **ESCALATION**	**HOST-REDUCE** **POLYTHEC-ACS**	2020	3429	ACS: 100% CCS: 0%	12 mo.	DAPT with prasugrel 5 mg vs. DAPT with prasugrel 10 mg	Death from all causes, MI, stent thrombosis, repeat revascularizations, stroke, bleeding BARC 2–5	Yes
		**TALOS-MI**	2021	2697	ACS: 100% CCS: 0%	12 mo.	DAPT with clopidogrel vs. DAPT with ticagrelor	Cardiovascular death, MI, stroke, bleeding BARC 2–5	Yes
		**TOPIC**	2017	646	ACS: 100% CCS: 0%	12 mo.	DAPT with clopidogrel vs. standard DAPT	Cardiovascular death, urgent revascularization, stroke, bleeding BARC 2–5	Yes

**Table 4:** Current guidelines recommendations of the European Society of Cardiology (ESC) and European Association for Cardio-Thoracic Surgery (EACTS) regarding guided antiplatelet therapy.

**2018 ESC/EACTS Guidelines on myocardial revascularization** [8]		
De-escalation of P2Y12 inhibitor treatment (e.g., with a switch from prasugrel or ticagrelor to clopidogrel) guided by platelet function testing may be considered as an alternative DAPT strategy, especially for ACS patients deemed unsuitable for 12-month potent platelet inhibition.	**IIb**	**B**
**2020 ESC Guidelines for the management of acute coronary syndromes in patients presenting without persistent ST-segment elevation** [3]		
De-escalation of P2Y12 receptor inhibitor treatment (e.g., with a switch from prasugrel or ticagrelor to clopidogrel) may be considered as an alternative DAPT strategy, especially for ACS patients deemed unsuitable for potent platelet inhibition. De-escalation may be completed unguided based on clinical judgment or guided by platelet function testing or CYP2C19 genotyping, depending on the patient’s risk profile and availability of respective assays.	**IIb**	**B**

## References

[1] Valgimigli M., Bueno H., Byrne R.A., Collet J.P., Costa F., Jeppsson A., Jüni P., Kastrati A., Kolh P., Mauri L. (2018). 2017 ESC focused update on dual antiplatelet therapy in coronary artery disease developed in collaboration with EACTS: The Task Force for dual antiplatelet therapy in coronary artery disease of the European Society of Cardiology (ESC) and of the European Association for Cardio-Thoracic Surgery (EACTS). Eur. Heart J..

[2] Knuuti J., Wijns W., Saraste A., Capodanno D., Barbato E., Funck-Brentano C., Prescott E., Storey R.F., Deaton C., Cuisset T. (2020). 2019 ESC Guidelines for the diagnosis and management of chronic coronary syndromes. Eur. Heart J..

[3] Collet J.P., Thiele H., Barbato E., Barthélémy O., Bauersachs J., Bhatt D.L., Dendale P., Dorobantu M., Edvardsen T., Folliguet T. (2021). 2020 ESC Guidelines for the management of acute coronary syndromes in patients presenting without persistent ST-segment elevation. Eur. Heart J..

[4] Gargiulo G., Esposito G., Avvedimento M., Nagler M., Minuz P., Campo G., Gragnano F., Manavifar N., Piccolo R., Tebaldi M. (2020). Cangrelor, Tirofiban, and Chewed or Standard Prasugrel Regimens in Patients with ST-Segment-Elevation Myocardial Infarction: Primary Results of the FABOLUS-FASTER Trial. Circulation.

[5] Valgimigli M., Gragnano F., Branca M., Franzone A., Baber U., Jang Y., Kimura T., Hahn J.Y., Zhao Q., Windecker S. (2021). P2Y12 inhibitor monotherapy or dual antiplatelet therapy after coronary revascularisation: Individual patient level meta-analysis of randomised controlled trials. BMJ.

[6] Gargiulo G., Valgimigli M., Capodanno D., Bittl J.A. (2017). State of the art: Duration of dual antiplatelet therapy after percutaneous coronary intervention and coronary stent implantation—Past, present and future perspectives. EuroIntervention.

[7] Gargiulo G., Windecker S., Vranckx P., Gibson C.M., Mehran R., Valgimigli M. (2016). A Critical Appraisal of Aspirin in Secondary Prevention: Is Less More?. Circulation.

[8] Neumann F.J., Sousa-Uva M., Ahlsson A., Alfonso F., Banning A.P., Benedetto U., Byrne R.A., Collet J.P., Falk V., Head S.J. (2019). 2018 ESC/EACTS Guidelines on myocardial revascularization. Eur. Heart J..

[9] Angiolillo D.J., Galli M., Collet J.P., Kastrati A., O’Donoghue M.L. (2022). Antiplatelet therapy after percutaneous coronary intervention. EuroIntervention.

[10] Corpataux N., Spirito A., Gragnano F., Vaisnora L., Galea R., Svab S., Gargiulo G., Zanchin T., Zanchin C., Siontis G.C.M. (2020). Validation of high bleeding risk criteria and definition as proposed by the academic research consortium for high bleeding risk. Eur. Heart J..

[11] Gragnano F., Spirito A., Corpataux N., Vaisnora L., Galea R., Gargiulo G., Siontis G.C.M., Praz F., Lanz J., Billinger M. (2021). Impact of clinical presentation on bleeding risk after percutaneous coronary intervention and implications for the ARC-HBR definition. EuroIntervention.

[12] Cesaro A., Taglialatela V., Gragnano F., Moscarella E., Fimiani F., Conte M., Barletta V., Monda E., Limongelli G., Severino S. (2020). Low-Dose Ticagrelor in Patients with High Ischemic Risk and Previous Myocardial Infarction: A Multicenter Prospective Real-World Observational Study. J. Cardiovasc. Pharmacol..

[13] Cesaro A., Gragnano F., Calabrò P., Moscarella E., Santelli F., Fimiani F., Patti G., Cavallari I., Antonucci E., Cirillo P. (2021). Prevalence and clinical implications of eligibility criteria for prolonged dual antithrombotic therapy in patients with PEGASUS and COMPASS phenotypes: Insights from the START-ANTIPLATELET registry. Int. J. Cardiol..

[14] Angiolillo D.J., Rollini F., Storey R.F., Bhatt D.L., James S., Schneider D.J., Sibbing D., So D.Y.F., Trenk D., Alexopoulos D. (2017). International Expert Consensus on Switching Platelet P2Y12 Receptor-Inhibiting Therapies. Circulation.

[15] Galli M., Franchi F., Rollini F., Angiolillo D.J. (2021). Role of platelet function and genetic testing in patients undergoing percutaneous coronary intervention. Trends Cardiovasc. Med..

[16] Moon J.Y., Franchi F., Rollini F., Rivas Rios J.R., Kureti M., Cavallari L.H., Angiolillo D.J. (2018). Role of genetic testing in patients undergoing percutaneous coronary intervention. Expert Rev. Clin. Pharmacol..

[17] Galli M., Franchi F., Rollini F., Cavallari L.H., Capodanno D., Crea F., Angiolillo D.J. (2021). Genetic testing in patients undergoing percutaneous coronary intervention: Rationale, evidence and practical recommendations. Expert Rev. Clin. Pharmacol..

[18] Sibbing D., Aradi D., Alexopoulos D., Ten Berg J., Bhatt D.L., Bonello L., Collet J.P., Cuisset T., Franchi F., Gross L. (2019). Updated Expert Consensus Statement on Platelet Function and Genetic Testing for Guiding P2Y 12 Receptor Inhibitor Treatment in Percutaneous Coronary Intervention. JACC Cardiovasc. Interv..

[19] Rodriguez F., Harrington R.A. (2021). Management of Antithrombotic Therapy after Acute Coronary Syndromes. N. Engl. J. Med..

[20] Perl L., Lerman-Shivek H., Rechavia E., Vaduganathan M., Leshem-Lev D., Zemer-Wassercug N., Dadush O., Codner P., Bental T., Battler A. (2014). Response to prasugrel and levels of circulating reticulated platelets in patients with ST-segment elevation myocardial infarction. J. Am. Coll. Cardiol..

[21] Pakala R., Waksman R. (2011). Currently available methods for platelet function analysis: Advantages and disadvantages. Cardiovasc. Revasc. Med..

[22] Jeong Y.H., Bliden K.P., Antonino M.J., Park K.S., Tantry U.S., Gurbel P.A. (2012). Usefulness of the VerifyNow P2Y12 assay to evaluate the antiplatelet effects of ticagrelor and clopidogrel therapies. Am. Heart J..

[23] Kong R., Trimmings A., Hutchinson N., Gill R., Agarwal S., Davidson S., Arcari M. (2015). Consensus recommendations for using the Multiplate^®^ for platelet function monitoring before cardiac surgery. Int. J. Lab. Hematol..

[24] Franchi F., Rollini F., Rivas J., Rivas A., Agarwal M., Briceno M., Wali M., Nawaz A., Silva G., Shaikh Z. (2020). Prasugrel Versus Ticagrelor in Patients With CYP2C19 Loss-of-Function Genotypes: Results of a Randomized Pharmacodynamic Study in a Feasibility Investigation of Rapid Genetic Testing. JACC Basic Transl. Sci..

[25] Roberts J.D., Wells G.A., le May M.R., Labinaz M., Glover C., Froeschl M., Dick A., Marquis J.F., O’Brien E., Goncalves S. (2012). Point-of-care genetic testing for personalisation of antiplatelet treatment (RAPID GENE): A prospective, randomised, proof-of-concept trial. Lancet.

[26] Chae H., Kim M., Koh Y.S., Hwang B.H., Kang M.K., Kim Y., Park H.I., Chang K. (2013). Feasibility of a microarray-based point-of-care CYP2C19 genotyping test for predicting clopidogrel on-treatment platelet reactivity. Biomed. Res. Int..

[27] Marziliano N., Notarangelo M.F., Cereda M., Caporale V., Coppini L., Demola M.A., Guidorossi A., Crocamo A., Pigazzani F., Boffetti F. (2015). Rapid and portable, lab-on-chip, point-of-care genotyping for evaluating clopidogrel metabolism. Clin. Chim. Acta.

[28] Notarangelo F.M., Maglietta G., Bevilacqua P., Cereda M., Merlini P.A., Villani G.Q., Moruzzi P., Patrizi G., Malagoli Tagliazucchi G., Crocamo A. (2018). Pharmacogenomic Approach to Selecting Antiplatelet Therapy in Patients with Acute Coronary Syndromes: The PHARMCLO Trial. J. Am. Coll. Cardiol..

[29] Angiolillo D.J., Capodanno D., Danchin N., Simon T., Bergmeijer T.O., Ten Berg J.M., Sibbing D., Price M.J. (2020). Derivation, Validation, and Prognostic Utility of a Prediction Rule for Nonresponse to Clopidogrel: The ABCD-GENE Score. JACC Cardiovasc. Interv..

[30] Rollini F., Franchi F., Angiolillo D.J. (2016). Switching P2Y12-receptor inhibitors in patients with coronary artery disease. Nat. Rev. Cardiol..

[31] Stone G.W., Witzenbichler B., Weisz G., Rinaldi M.J., Neumann F.J., Metzger D.C., Henry T.D., Cox D.A., Duffy P.L., Mazzaferri E. (2013). Platelet reactivity and clinical outcomes after coronary artery implantation of drug-eluting stents (ADAPT-DES): A prospective multicentre registry study. Lancet.

[32] Price M.J., Berger P.B., Teirstein P.S., Tanguay J.F., Angiolillo D.J., Spriggs D., Puri S., Robbins M., Garratt K.N., Bertrand O.F. (2011). Standard- vs high-dose clopidogrel based on platelet function testing after percutaneous coronary intervention: The GRAVITAS randomized trial. JAMA.

[33] Trenk D., Stone G.W., Gawaz M., Kastrati A., Angiolillo D.J., Müller U., Richardt G., Jakubowski J.A., Neumann F.J. (2012). A randomized trial of prasugrel versus clopidogrel in patients with high platelet reactivity on clopidogrel after elective percutaneous coronary intervention with implantation of drug-eluting stents: Results of the TRIGGER-PCI (Testing Platelet Reactivity in Patients Undergoing Elective Stent Placement on Clopidogrel to Guide Alternative Therapy with Prasugrel) study. J. Am. Coll. Cardiol..

[34] Cayla G., Cuisset T., Silvain J., Leclercq F., Manzo-Silberman S., Saint-Etienne C., Delarche N., Bellemain-Appaix A., Range G., El Mahmoud R. (2016). Platelet function monitoring to adjust antiplatelet therapy in elderly patients stented for an acute coronary syndrome (ANTARCTIC): An open-label, blinded-endpoint, randomised controlled superiority trial. Lancet.

[35] Sibbing D., Aradi D., Jacobshagen C., Gross L., Trenk D., Geisler T., Orban M., Hadamitzky M., Merkely B., Kiss R.G. (2017). Guided de-escalation of antiplatelet treatment in patients with acute coronary syndrome undergoing percutaneous coronary intervention (TROPICAL-ACS): A randomised, open-label, multicentre trial. Lancet.

[36] Zheng Y.Y., Wu T.T., Yang Y., Hou X.G., Gao Y., Chen Y., Yang Y.N., Li X.M., Ma X., Ma Y.T. (2020). Personalized antiplatelet therapy guided by a novel detection of platelet aggregation function in stable coronary artery disease patients undergoing percutaneous coronary intervention: A randomized controlled clinical trial. Eur. Heart J. Cardiovasc. Pharmacother..

[37] Claassens D.M.F., Vos G.J.A., Bergmeijer T.O., Hermanides R.S., van’t Hof A.W.J., van der Harst P., Barbato E., Morisco C., Tjon Joe Gin R.M., Asselbergs F.W. (2019). A Genotype-Guided Strategy for Oral P2Y 12 Inhibitors in Primary PCI. N. Engl. J. Med..

[38] Pereira N.L., Farkouh M.E., So D., Lennon R., Geller N., Mathew V., Bell M., Bae J.H., Jeong M.H., Chavez I. (2020). Effect of Genotype-Guided Oral P2Y12 Inhibitor Selection vs Conventional Clopidogrel Therapy on Ischemic Outcomes after Percutaneous Coronary Intervention: The TAILOR-PCI Randomized Clinical Trial. JAMA.

[39] Tuteja S., Glick H., Matthai W., Nachamkin I., Nathan A., Monono K., Carcuffe C., Maslowski K., Chang G., Kobayashi T. (2020). Prospective CYP2C19 Genotyping to Guide Antiplatelet Therapy Following Percutaneous Coronary Intervention: A Pragmatic Randomized Clinical Trial. Circ. Genom. Precis Med..

[40] Cuisset T., Deharo P., Quilici J., Johnson T.W., Deffarges S., Bassez C., Bonnet G., Fourcade L., Mouret J.P., Lambert M. (2017). Benefit of switching dual antiplatelet therapy after acute coronary syndrome: The TOPIC (timing of platelet inhibition after acute coronary syndrome) randomized study. Eur. Heart J..

[41] Kim H.S., Kang J., Hwang D., Han J.K., Yang H.M., Kang H.J., Koo B.K., Rhew J.Y., Chun K.J., Lim Y.H. (2020). Prasugrel-based de-escalation of dual antiplatelet therapy after percutaneous coronary intervention in patients with acute coronary syndrome (HOST-REDUCE-POLYTECH-ACS): An open-label, multicentre, non-inferiority randomised trial. Lancet.

[42] Kim C.J., Park M.W., Kim M.C., Choo E.H., Hwang B.H., Lee K.Y., Choi Y.S., Kim H.Y., Yoo K.D., Jeon D.S. (2021). Unguided de-escalation from ticagrelor to clopidogrel in stabilised patients with acute myocardial infarction undergoing percutaneous coronary intervention (TALOS-AMI): An investigator-initiated, open-label, multicentre, non-inferiority, randomised trial. Lancet.

[43] Galli M., Benenati S., Capodanno D., Franchi F., Rollini F., D’Amario D., Porto I., Angiolillo D.J. (2021). Guided versus standard antiplatelet therapy in patients undergoing percutaneous coronary intervention: A systematic review and meta-analysis. Lancet.

[44] Galli M., Benenati S., Franchi F., Rollini F., Capodanno D., Biondi-Zoccai G., Vescovo G.M., Cavallari L.H., Bikdeli B., Ten Berg J. (2022). Comparative effects of guided vs. potent P2Y12 inhibitor therapy in acute coronary syndrome: A network meta-analysis of 61,898 patients from 15 randomized trials. Eur. Heart J..

[45] Kuno T., Fujisaki T., Shoji S., Sahashi Y., Tsugawa Y., Iwagami M., Takagi H., Briasoulis A., Deharo P., Cuisset T. (2022). Comparison of Unguided De-Escalation versus Guided Selection of Dual Antiplatelet Therapy after Acute Coronary Syndrome: A Systematic Review and Network Meta-Analysis. Circ. Cardiovasc. Interv..

[46] Patti G., Grisafi L., Spinoni E.G., Rognoni A., Mennuni M. (2022). Safety and Efficacy of Selective, Clopidogrel-Based Strategies in Acute Coronary Syndrome: A Study-Level Meta-analysis. Thromb. Haemost..

[47] Galli M., Ortega-Paz L., Franchi F., Rollini F., Angiolillo D.J. (2022). Precision medicine in interventional cardiology: Implications for antiplatelet therapy in patients undergoing percutaneous coronary intervention. Pharmacogenomics.

